# Adjunctive multimodal extracorporeal support in severe septic shock with multi-organ failure: a case report

**DOI:** 10.3389/fmed.2026.1751691

**Published:** 2026-01-29

**Authors:** Pedja Kovacevic, Tanja Knezevic, Danica Momcicevic, Milka Jandric, Ognjen Djakovic, Biljana Zlojutro, Sasa Dragic

**Affiliations:** 1 Medical Intensive Care Unit, University Clinical Centre of the Republic of Srpska, Banja Luka, Bosnia and Herzegovina; 2 Faculty of Medicine, University of Banja Luka, Banja Luka, Bosnia and Herzegovina

**Keywords:** CRRT, hemoadsorption, multi-organ failure, plasma exchange, septic shock

## Abstract

Septic shock is a life-threatening condition that frequently progresses to multi-organ dysfunction despite optimal standard-of-care therapies. Extracorporeal blood purification (EBP) techniques have gained attention as adjunctive strategies aimed at modulating hyperinflammation and supporting failing organs. We describe the case of a 64-year-old woman with septic shock, likely secondary to urosepsis, complicated by acute respiratory failure, acute kidney injury, acute liver failure, and cytopenias. Initial management with fluid resuscitation, empirical broad-spectrum antibiotics, corticosteroids, and non-invasive ventilation proved insufficient. Given profound hyperinflammation (IL-6: 47,089 pg./mL) and severe metabolic derangements, continuous veno-venous hemodiafiltration (CVVHDF) with hemoadsorption was initiated, resulting in rapid IL-6 reduction and hemodynamic stabilization. As her clinical course evolved, a multimodal EBP approach, including sequential hemoadsorption devices, therapeutic plasma exchange, and the double plasma molecular adsorption system (DPMAS), was employed to support renal and hepatic function, at one point requiring two separate extracorporeal circuits operating concurrently for different EBP modalities. Progressive respiratory failure necessitated invasive mechanical ventilation, prone positioning, and subsequent tracheostomy. The patient ultimately recovered, with restoration of hepatic and renal function, resolution of cytopenias, and successful weaning from mechanical ventilation. This case underscores the potential role of adjunctive multimodal EBP in refractory septic shock with severe multi-organ failure. Although clinical improvement paralleled biomarker reduction, causal inference is limited by the concurrent use of multiple therapies. Robust randomized studies are needed to clarify optimal protocols, timing, and patient selection for combined extracorporeal strategies in critical sepsis.

## Introduction

Septic shock is a life-threatening condition characterized by a dysregulated host response to infection, often leading to multi-organ failure. Despite advances in antimicrobial therapy and organ support, the mortality rate remains high (1). Conventional treatments may prove inadequate in cases involving severe hyperinflammation, acute kidney injury, and liver failure, leading to the consideration of extracorporeal blood purification techniques as additional therapies. Recently, various modalities, including continuous renal replacement therapy (CRRT), hemoadsorption, therapeutic plasma exchange (TPE), and artificial liver support systems, have been studied as strategies to reduce overwhelming systemic inflammation, support failing organs, and improve outcomes in critically ill patients with sepsis (2, 3). We describe the case of a 64-year-old woman with septic shock, likely secondary to urosepsis, complicated by profound respiratory, renal, hepatic, and hematologic dysfunction. She underwent a multimodal extracorporeal support strategy that included continuous veno-venous hemodiafiltration (CVVHDF) with hemoadsorption, TPE, and the double plasma molecular adsorption system (DPMAS). This case underscores both the potential benefits and the inherent challenges of integrating multiple extracorporeal support modalities into the comprehensive management of severe and refractory septic shock.

## Case report

### Patient information and history

A 64-year-old woman with a history of asthma and obesity was admitted to the Department of Medical Intensive Care Medicine (MICU) with primary diagnoses of septic shock, acute liver failure, and acute respiratory failure. The patient had a recently treated urinary tract infection that likely represented the primary infectious focus, which had shown inadequate response to initial antibiotic therapy. Several days before admission, symptoms recurred, and this suboptimally managed infection likely facilitated systemic dissemination of a multidrug-resistant strain, culminating in septic shock and multiorgan failure. On the day of admission, she developed sudden dyspnea and wheezing. Despite receiving her regular asthma medication and intensive inhalation therapy, her condition did not improve, and she presented to the Emergency Department. Non-invasive mechanical ventilation (NIV) was initiated immediately, and after consultation with an intensivist, she was admitted to the MICU.

### Clinical findings, laboratory and imaging results on admission

On admission to MICU (day 1), the patient was conscious but anxious, tachydyspneic, and on NIV (FiO₂ 1.0, PEEP 5, PS 0), achieving peripheral oxygen saturation (SpO₂) of 95%. She was tachycardic (130 bpm), hypotensive (100/75 mmHg), and hypothermic (35.3 °C). On auscultation, diffuse wheezing accompanied by bilateral coarse crackles was noted. Cardiac examination revealed rapid, regular heart sounds with soft intensity and no appreciable murmur. On examination, the abdomen was markedly distended up to the lower chest level but remained soft and non-tender on palpation. Bilateral pretibial edema was noted. Venous blood gas analysis revealed: pH 7.11, pCO₂ 4.5 kPa, pO₂ 6.9 kPa, SpO₂ 71%, HCO₃^−^ 10.8 mmol/L, base excess - 18.7 mmol/L, and lactate 11.9 mmol/L. Laboratory investigations demonstrated elevated inflammatory markers, thrombocytopenia, azotemia, elevated transaminases, and coagulopathy. Chest radiography showed bilateral pulmonary consolidations with pleural effusions. Abdominal and pelvic CT revealed bilateral pleural effusions (5.6 cm on the right, 3.6 cm on the left, AP diameter), atelectatic parenchymal changes, decreased renal cortical enhancement with blurred corticomedullary differentiation, and perirenal fat stranding. Free fluid was noted perihepatically, perisplenically, and extending into the pelvis.

### Diagnosis and differential diagnosis

Our primary diagnosis was septic shock with multiorgan failure, most likely resulting from a previously inadequately treated urinary tract infection, but during the course of treatment, several differential diagnoses were considered. Thrombotic thrombocytopenic purpura (TTP) was initially suspected based on peripheral smear findings but was later excluded once delayed ADAMTS13 results became available, as the test could not be performed on-site. Viral hepatitis and hemorrhagic fever were also ruled out through negative serologies. Myelodysplastic syndrome was initially considered; however, upon review of the bone marrow biopsy, hematologic malignancy was ultimately excluded. In the following text, the approach to potential differential diagnoses is discussed, while the patient was simultaneously managed for septic shock with multiorgan dysfunction, receiving appropriate etiological, symptomatic, and supportive therapy.

### Therapeutic management, clinical course, and outcomes

Initial therapy in MICU (day 1) included intravenous crystalloids and colloids with electrolyte correction, ulcer prophylaxis, corticosteroid therapy (methylprednisolone), diuretics, and inhaled bronchodilator therapy. Empirical triple antibiotic therapy was initiated, consisting of vancomycin 1 g twice daily, meropenem 1 g three times daily, and azithromycin 500 mg once daily. Guided by rotational thromboelastometry (ROTEM) due to coagulopathy, the patient received transfusion of blood components, including packed red blood cells, platelets, cryoprecipitate, and fresh frozen plasma, as well as antifibrinolytic therapy. Mechanical prophylaxis for deep vein thrombosis (DVT) was instituted. Due to severe hyperinflammation (IL-647089 pg./mL), metabolic acidosis, and acute kidney injury (AKI), a central venous catheter was placed, and oXiris® based CVVHDF was initiated without systemic anticoagulation. The decision to initiate oXiris® therapy upon admission was based on a strong clinical suspicion of endotoxin-mediated shock, supported by the patient’s clinical presentation and laboratory findings, including markedly elevated IL-6 and lactate levels. oXiris®-based CVVHDF was performed using a blood flow rate of 150–200 mL/min. The prescribed CRRT dose was 25–30 mL/kg/h, delivered in continuous veno-venous hemodiafiltration mode, with dialysate flow rates of approximately 20–25 mL/kg/h. IL-6 levels rapidly decreased from 47,089 to 1,260 pg./mL within 11 h of therapy. This was accompanied by hemodynamic stabilization and a reduction in lactate from 11.9 to 6.7 mmol/L. On days 2, despite NIV, respiratory failure progressed with altered consciousness, necessitating intubation and initiation of controlled mechanical ventilation, sedation, analgesia, and neuromuscular blockade. Norepinephrine infusion (0.1 μg/kg/min) was started and titrated to maintain arterial pressure. Due to anuria and persistent metabolic acidosis, CVVHDF without anticoagulation and hemoadsorption (oXiris®) was continued using the same prescribed parameters. The switch from the oXiris® hemoadsorbent to the Jafron® HA330-II filter (days 3 and 4) was clinically based on signs of acute hepatocellular dysfunction [alanine aminotransferase (ALT) 3,060 U/L], and the treatment was performed using the same CVVHDF settings as the oXiris®-based therapy, without anticoagulation.

**Figure 1:**
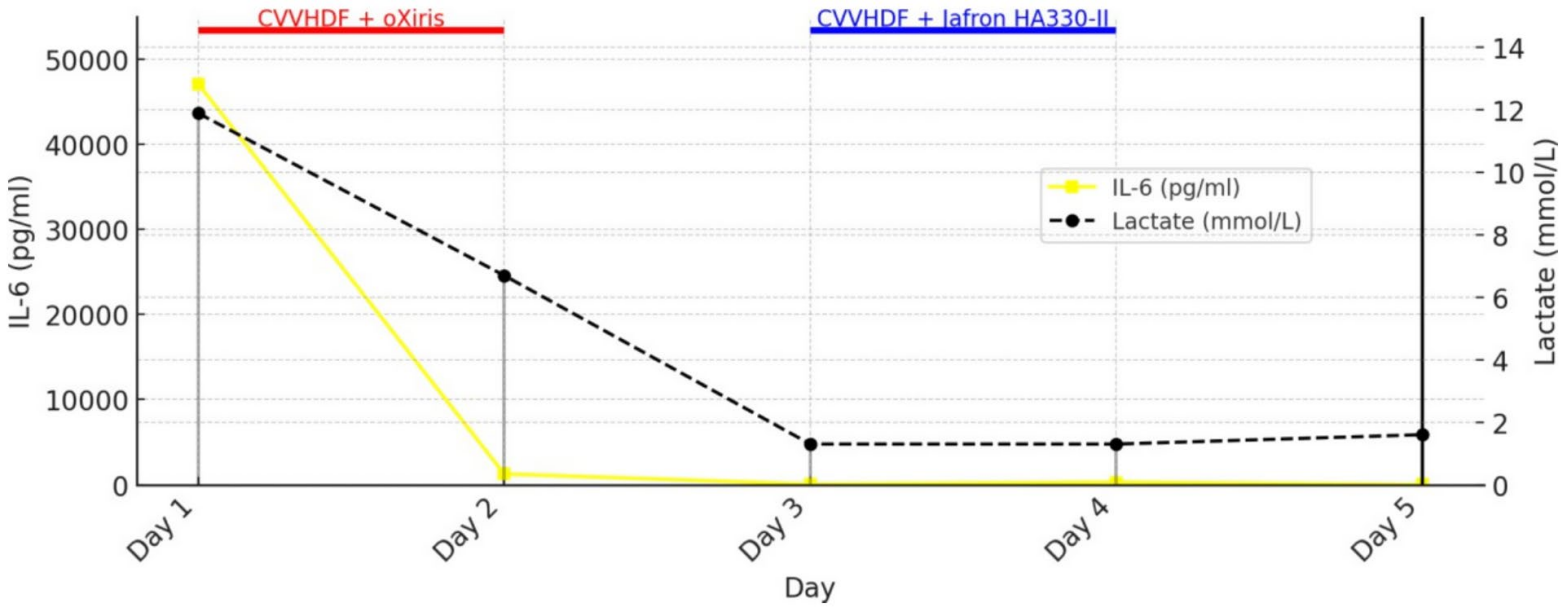
Trends in IL-6 and lactate during treatment with oXiris^®^ and Jafron^®^ HA330-II hemoadsorbents.

**Figure 2:**
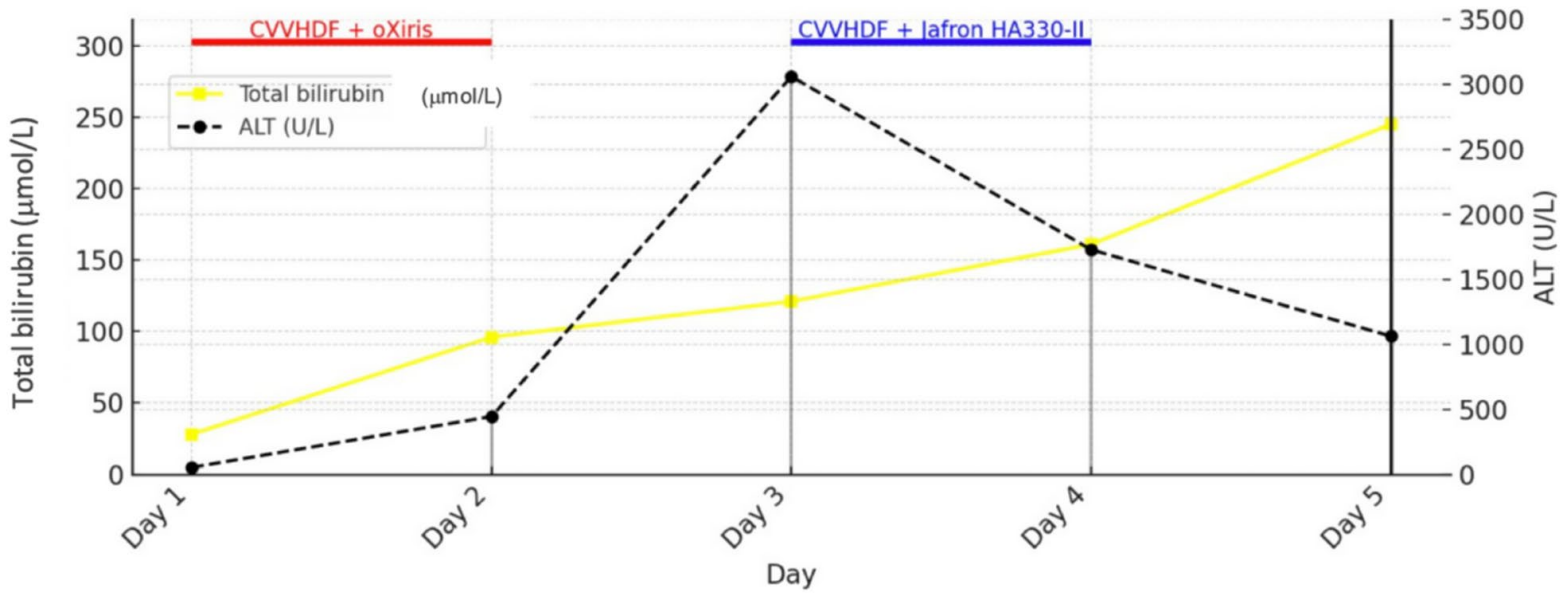
Trends in total bilirubin and ALT during treatment with oXiris^®^ and Jafron^®^ HA330-II hemoadsorbents.

Figure 1 shows the trends of IL-6 and lactate in relation to the use of Oxiris and Jafron® HA330-II hemoadsorbents (rapid decrease of IL-6 and lactate after 2 cycles of hemoadsorption with the oXiris® filter), while Figure 2 shows the trends of total bilirubin and ALT in relation to the use of the same hemoadsorbents (rapid decrease of ALT after 2 cycles of hemoadsorption with the Jafron® HA330-II filter, with no significant effect on total bilirubin).

Given the acute liver failure and rising bilirubin levels, the DPMAS Jafron® adsorption system was applied on five occasions (days five to ten) due to the development of marked hyperbilirubinemia and deterioration of liver function despite previous therapeutic modalities. DPMAS therapy was performed using a blood flow rate of 80–120 mL/min, with a plasma separation rate of approximately 20–30% of the blood flow. The system consisted of sequential plasma adsorption cartridges (HA330-II and BS330) according to the manufacturer’s recommendations. Each treatment session lasted approximately 2–4 h, with the number of sessions guided by clinical response and trends in liver function parameters. Given severe ARDS, the patient underwent multiple prone positioning sessions and bronchoscopy for airway clearance. Microbiological testing revealed *Enterococcus* spp. in urine culture and *Staphylococcus aureus* and *Streptococcus pneumoniae* in tracheal aspirates, all susceptible to the empiric antibiotic regimen. Serology for viral hemorrhagic fever was negative. Peripheral blood smear revealed anemia and thrombocytopenia, accompanied by biochemical abnormalities - hyperbilirubinemia, elevated LDH, hypofibrinogenemia, and elevated D-dimer, suggestive of TTP. TPE was initiated on day 11 and performed in 13 sessions, each exchanging 5 L of fresh frozen plasma, in combination with high-dose corticosteroids (methylprednisolone 500 mg twice daily for three days). TPE was initiated on day 11 and performed in 13 sessions, each exchanging approximately 5 L of fresh frozen plasma. Treatments were delivered at a blood flow rate of 80–120 mL/min, corresponding to approximately 1–1.5 plasma volumes per session. Although TTP was initially suspected, given the presence of a cytokine storm, multiorgan dysfunction, elevated bilirubin, and coagulopathy, plasma exchange was considered to offer a greater potential benefit than harm and may have served as a ‘bridge’ to spontaneous liver recovery. Despite initial suspicion, ADAMTS13 testing later excluded TTP.

**Figure 3:**
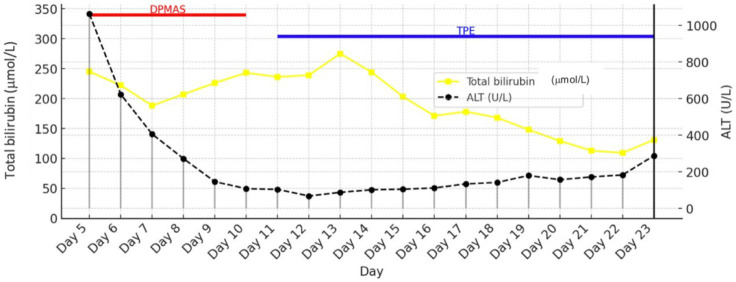
Trends in total bilirubin and ALT during DPMAS therapy.

The effects of DPMAS and TPE therapy are shown in Figure 3 (effect of DPMAS on indirect ALT reduction and effect of TPE on bilirubin reduction).

A bone marrow biopsy performed on day 14 for persistent cytopenias initially suggested myelodysplastic syndrome, but review in a reference laboratory confirmed reactive erythroid hyperplasia with secondary megaloblastic changes, without evidence of hematologic malignancy. A percutaneous tracheostomy was performed on day 14 due to prolonged mechanical ventilation and the inability to wean the patient. CVVHDF was maintained until recovery of renal function on day 38 performed in parallel with hemoadsorption therapy (Oxiris® and Jafron® HA330-II filters), while DPMAS sessions and TPE were conducted simultaneously on a separate extracorporeal circuit. After sedation was discontinued, the patient gradually regained consciousness and was successfully weaned from mechanical ventilation. Decannulation occurred on day 63. Persistent anemia was managed with multiple transfusions and intravenous iron therapy for iron-deficiency anemia. Renal function recovered, diuresis was restored, and the urinary catheter was removed. Physical rehabilitation was initiated during the MICU stay. On hospital day 97, the patient was discharged to a rehabilitation center in stable condition, hemodynamically stable, with adequate respiratory function, fully conscious, and mobilized.

## Discussion

Septic shock produces a dysregulated host response that frequently leads to life-threatening multi-organ failure (1). In this case, a poorly and insufficiently treated urinary tract infection was identified as the most likely source of infection, and massive activation of the host inflammatory response resulted in impaired microvascular perfusion, endothelial dysfunction, and the development of multiorgan failure, which are characteristic features of severe sepsis. When conventional therapy, such as appropriate antimicrobial treatment, fluid resuscitation, and vasopressors, is insufficient, extracorporeal blood purification (EBP) techniques are increasingly considered as adjunctive options to modulate excessive inflammation and support failing organs (2, 3). Commonly used EBP modalities in intensive care include CRRT with adsorptive membranes, hemoadsorption cartridges, TPE, and artificial liver support systems like the DPMAS. These techniques aim to remove circulating cytokines, endotoxin, bilirubin, and other toxic mediators or to replace dysfunctional plasma components, potentially blunting the “cytokine storm” and bridging patients through the acute phase (4–9). In the presented patient, initiation of CVVHDF with a cytokine-adsorbing membrane (e.g., oXiris®) was associated with a rapid decline in IL-6 levels and subsequent clinical stabilization. This temporal pattern, early reduction in inflammatory biomarkers followed by hemodynamic improvement, has been described in multiple observational studies and small clinical trials of oXiris® and other adsorptive devices, although the studies differ in design and endpoints (10, 11). Recent pilot studies and case series report decreases in IL-6, CRP, and vasopressor requirements after early use of adsorptive hemofilters; some pilot randomized trials are ongoing. However, high-quality randomized evidence demonstrating a clear mortality benefit remains limited (12). Therefore, although the biological plausibility and temporal association in this case are compelling, causality cannot be definitively established. TPE was initially applied due to suspected TTP. While TPE remains the treatment choice for TTP, its role in sepsis is more debated. Several systematic reviews and meta-analyses of observational studies suggest that adjunctive TPE may speed up organ recovery and, in some pooled analyses, be associated with lower short-term mortality in specific adult groups; however, heterogeneity among studies, bias risk, and variable protocols prevent firm conclusions. Ongoing randomized trials aim to better define patient selection, timing, and overall clinical benefit (13–15). Clinicians should reserve TPE for specific indications (e.g., confirmed TTP) or consider it as a rescue therapy within a multidisciplinary decision-making process. DPMAS (often used alone or sequentially with low-volume plasma exchange) has been reported to reduce bilirubin and other toxins and to improve laboratory markers in cases of acute or acute-on-chronic liver failure. Although evidence for DPMAS is stronger in liver failure literature than specifically in sepsis, most studies are observational or non-randomized; nonetheless, DPMAS can serve as a bridging technique while awaiting hepatic recovery or definitive therapy (16–18). In the observed case, DPMAS was part of a multimodal approach aimed at controlling systemic toxicity and supporting failing liver function. Combining multiple extracorporeal therapies poses practical and safety challenges that must be actively managed, including anticoagulation strategies (especially in coagulopathic patients), vascular access logistics, sequencing of modalities, and the risk of unintended removal of drugs (notably some antimicrobials) or essential plasma components (19). Recent reports note clinically significant adsorption of certain antibiotics and antipathogen agents by highly adsorptive filters, highlighting the need for dose adjustments and therapeutic drug monitoring where practical. Point-of-care coagulation testing (e.g., ROTEM), careful transfusion strategies, and close multidisciplinary coordination can reduce some risks but cannot eliminate them (20).

### Strengths and limitations

Strengths of this case report include the detailed real-world description of a multimodal extracorporeal blood purification strategy, with sequential and concurrent use of multiple devices guided by evolving organ dysfunction. Additional strengths are the comprehensive reporting of technical parameters and the parallel presentation of clinical and laboratory trends, which provide practical insight into complex decision-making in refractory septic shock with multi-organ failure. It is important to emphasize the limitations in the current literature and in our single-case observations. Most supportive studies are observational, single-center, or retrospective with variable patient populations and device parameters. Until stronger evidence emerges, the use of EBP should be individualized, discussed within a multidisciplinary team, and closely monitored through systematic clinical and laboratory assessments.

## Conclusion

This case supports the concept that a multimodal extracorporeal approach, combining CRRT with adsorptive membranes, hemoadsorption cartridges, DPMAS, and TPE when indicated, can be integrated into a comprehensive strategy for selected patients with septic shock and severe multi-organ dysfunction. The rapid IL-6 decrease and clinical stabilization observed here are consistent with prior observational reports of biomarker and hemodynamic improvement following extracorporeal interventions, but causal inference is limited by concomitant therapies and the observational nature of the evidence. Well-designed randomized studies and harmonized reporting are urgently needed to determine efficacy, safety, and optimal protocols for these adjunctive therapies.

### Take-home message

CVVHDF + oXiris® was started in cases of septic shock complicated by AKI, characterized by anuria or severe oliguria, rising nitrogen waste products, and ongoing metabolic acidosis, along with clinical and lab signs of hyperinflammation (e.g., high inflammatory markers) and endotoxemia. Therapy was stopped once metabolic stability was achieved, kidney function stabilized, and inflammation decreased.

CVVHDF + Jafron® HA330-II was initiated when systemic inflammation continued despite initial treatment, especially with acute liver injury, indicated by significant rises in aminotransferases and worsening liver function. Treatment was ceased after improvements in inflammation and liver enzyme trends.

DPMAS (Jafron® adsorption system) was started in patients with acute liver failure characterized by severe hyperbilirubinemia, worsening coagulopathy, and clinical signs of liver dysfunction. Therapy was halted after bilirubin levels decreased sustainably and liver and coagulation functions stabilized.

Therapeutic plasma exchange (TPE) was used in refractory acute liver failure with ongoing hyperbilirubinemia, coagulopathy, and thrombocytopenia even after prior extracorporeal support. TPE was discontinued after liver function tests, coagulation, and platelet counts improved.

## Data Availability

The raw data supporting the conclusions of this article will be made available by the authors, without undue reservation.
